# Diet quality in medical trainees: a cross-sectional assessment comparing medical students and primary care residents using the Rapid Eating Assessment for Participants—shortened version

**DOI:** 10.1186/s40795-024-00899-x

**Published:** 2024-07-25

**Authors:** Birgit Khandalavala, Stephanie Emig, Mira Yousef, Jenenne Geske

**Affiliations:** 1 University of Nebraska Medical Center, Omaha, NE USA; 2 Nebraska Medicine, Omaha, NE USA

**Keywords:** Diet quality, Medical profession trainees, Health outcomes, BMI, REAP-S

## Abstract

**Background:**

The diet quality of the US population is significantly unhealthy, with critical long-term implications for the nation’s health. A few studies have explored diet quality in the future primary care workforce. This cross-sectional study quantifies the diet quality of medical students and primary care residents at a Midwestern college of medicine in the United States.

**Methods:**

250 medical students and 148 primary care residents were surveyed electronically utilizing the Rapid Eating Assessment for Participants—Shortened Version (REAP-S). The survey consists of 13 questions that can be scored on a scale from 1 to 3, along with 3 questions that are not scored. The average REAP-S score for a US omnivorous diet is 32 (range 13 to 39) with higher scores indicating a higher quality diet. We obtained average REAP-S scores for all respondents and descriptive summary statistics for individual REAP-S items. Students’ REAP-S total scores were compared to those of residents and the interaction between student/resident status and BMI category on REAP-S total scores were analyzed using analyses of variance. Differences between students and residents on BMI categories and other outcomes (individual REAP-S items, pandemic dietary and weight changes) were analyzed using Chi-Square Tests of Independence or Fisher’s Exact Tests.

**Results:**

Medical students (*n* = 99; 39.6% response rate) had a significantly higher mean REAP-S score (30.5, SD = 3.9) than primary care residents (*n* = 72; 49% response rate) (mean = 28.6, SD = 3.9; *p* = 0.006). Total mean REAP-S scores were significantly higher for those with BMI < 25 (mean = 30.8, SD = 3.7) than for those with BMI > = 25 (mean = 28.3, SD = 4.0; *p* < 0.001). There was not a statistically significant interaction between role (student vs. resident) and BMI category on total REAP-S scores (*p* = 0.39). Most respondents (89.3%) indicated that they were willing to improve their diet.

**Conclusion:**

Our data suggest that the diet quality of surveyed medical students and primary care residents, as quantified by the REAP-S, is suboptimal. Early detection and improvement of diet quality may be necessary for our medical profession trainees to avert potential long-term adverse cardiometabolic health outcomes, and to optimize the health of our future primary care workforce.

## Introduction

The critical role diet quality plays in many non-communicable diseases, including cancer and mental health, is becoming evident [1, 2]. Poor diet quality, along with lack of physical activity, has emerged as a crucial driver of cardiovascular health outcomes. Various reports emphasize the national and global urgency to better understand diet quality, as well as to analyze the factors that contribute to poor diet quality and identify ways to improve diet [3]. A healthy diet, particularly in early adult life, can extend the “health span” of the general population. Additionally, members of our future healthcare workforce, who will be at the forefront of managing increasing chronic disease burdens due to lifestyle factors, need to understand and manage their own diet quality [4].

Diet quality is broadly defined as a dietary pattern or an indicator of variety across the main food groups as recommended in national nutrition guidelines. It consists of an overall assessment of the foods consumed rather than any single nutrient or food [5, 6]. The terms “diet quality” and “dietary quality” have been used interchangeably in publications referring to the overall quality of a diet. In this study, the authors will use the term “diet quality.”

The concept of diet quality is relatively new and evolving; overall diet quality has been noted to be more correlated with health than any individual macronutrient or isolated food items. However, a clear understanding of dietary metrics is essential for expanding research around this topic [5–8]. Poor diet quality has emerged as one of the most important risk factors for mortality and is associated with 11 million deaths and approximately 50% of cardiovascular deaths globally [9].

In order to quantify diet quality, Diet Quality Indices (DQIs) have been developed. These assessment tools utilize matrices that score food and/or nutrient intakes by frequency or estimation of consumption and according to how closely they align with dietary guidelines. [7]. Some indices include additional lifestyle factors or other items that may not be scored [5]. The Healthy Eating Index (HEI) is a premier dietary quality index that is regularly updated and aligns with the latest United States (US) dietary guidelines. However, it is rather lengthy, with a 15-minute completion time, and is used primarily in research settings [10]. The Rapid Eating Assessment for Participants (REAP) was developed as a diet quality screening tool to aid nutritional counseling in clinical practice where time is often constrained [11]. A shortened version of this dietary index was subsequently developed (REAP-S) and validated in a group of medical trainees [12] and has been found to correlate with cardiometabolic outcomes [13, 14].

A wide variety of complex factors contribute to the quality of foods consumed by an individual, including socio-economic and psychological factors [15]. Little is known about diet quality in US medical students or primary care residents. There is some research that examined nutrient intake or diet quality of medical students in the context of culinary nutrition courses or other nutrition-focused education [16–19]. Other studies examined specific dietary components rather than overall diet quality [20–22]. The aim of the current study is to examine and compare overall diet quality, as measured by the REAP-S, of primary care residents and medical students, independent of targeted nutritional educational endeavors. Additionally, we examine how diet quality differs based on body mass index (BMI). Our hypotheses were:

1. Overall diet quality for primary care residents would be lower than that of medical students.
2. Those with a body mass index (BMI) > = 25 would have poorer diet quality.
3. The relationship between BMI and diet quality would not differ depending on whether the respondent was a resident or a student.

## Methods

### Study design

This cross-sectional, single-center survey study was conducted at an academic medical center in the midwestern US.

### Participants and recruitment

The study population consisted of two groups. The first group consisted of all medical students officially enrolled in June 2020 (*n* = 250). This group included students who were just finishing their 1st or 2nd years of medical school. Fourth-year students had already graduated, and incoming 1st year students had not yet matriculated. The second group consisted of all residents who were training in family medicine and internal medicine in May-June, 2021 (*n* = 148). All potential participants received an email invitation to participate in the study. Clearance to conduct the survey was granted from the Institutional Review Board (IRB #492-20-EX and IRB #0217-21-EX) of the affiliated university medical center and from the dean of the medical school. Informed consent was given at the time of the survey.

### Instruments

The researchers compiled a survey to address the research objectives. Questions included some demographic information along with questions about respondents’ height and weight.

Diet quality was assessed using the Rapid Eating Assessment for Participants—Shortened Version (REAP-S). The REAP-S consists of 16 questions about food consumption. The first 13 questions ask participants to recall their eating behavior, dietary patterns, and types and quantities of food eaten during the prior week. Questions 14–16 collect information on current cooking and eating habits (eating at home versus eating out) and willingness to improve eating patterns. Diet quality was quantified by scoring questions 1–13 on a 3-point scale: Usually/Often (1 point), Sometimes (2 points), and Rarely/Never or Does Not Apply to Me (3 points). Questions 14–16 were not included in the total score. Total possible scores range from 13 to 39, with higher scores indicative of better diet quality. Based on the average omnivorous US diet, the developers of the REAP-S determined that a score > = 32 can be considered a “healthier” diet [14].

The REAP-S has been validated with dietary intakes in medical students. It correlates with the much longer Healthy Eating Index and other well-documented markers of diet quality and cardiometabolic health [11, 12, 14, 23]. The REAP-S does not need trained staff to administer or interpret results and has negligible costs associated with its use.

### Survey procedure

All enrolled medical students and primary care residents were emailed an invitation to participate in the study with instructions for accessing the web-based survey. Surveys remained open for two weeks. A reminder email was sent at the end of the first week. Participants were asked to reference an average week when answering the survey questions. Responses were captured anonymously in a secure database.

### Data analysis

Responses to height and weight questions were used to calculate body mass index (BMI) using the following formula:

$$BMI=703* \frac{weight in pounds}{{\left(height in inches\right)}^{2}}$$

We dichotomized BMI into two groups, those with BMI < 25 kg/m^2^ (normal weight and below) and those with BMI > = 25 kg/m^2^ (overweight and obese).

We obtained average REAP-S scores for all respondents and descriptive summary statistics for individual REAP-S items. Students’ REAP-S total scores were compared to those of residents and the interaction between student/resident status and BMI category on REAP-S total scores were analyzed using analyses of variance. Differences between students and residents on BMI categories and other outcomes (individual REAP-S items, pandemic dietary and weight changes) were analyzed using Chi-Square Tests of Independence (when minimum expected frequencies were met) or Fisher’s Exact Tests (when expected frequencies were too small for Chi-Square tests). All analyses were conducted using SPSS v28.0. An alpha level of 0.05 was used to assess statistical significance.

## Results

Complete responses on all REAP-S items were received from 171 participants (43% response rate); 99 were medical students (out of 250; 39.6% response rate) and 72 were residents (out of 148; 48.6% response rate). Of the residents who responded to the survey, 47% were women and the average age was 29.19 years. Information on race/ethnicity was not collected. Gender, age and race/ethnicity were not collected from student respondents. However, in Spring 2020, 79.7% of the students enrolled in the College of Medicine were White non-Hispanic, 45.3% were women and 88.1% were between the ages of 22-29 [24]. Those who did not have complete REAP-S responses (3 students and 4 residents) were excluded from all analyses. The average REAP-S score for all respondents was 29.7 (SD = 4.0).

A total of 54.7% of respondents reported a BMI < 25, while 45.3% reported a BMI > = 25. Students (59.2%) tended to be more likely than residents (48.6%) to report a BMI < 25, although results of a Chi Square Test of Independence revealed that this difference was not statistically significant (*p* = 0.17).

A factorial analysis of variance was used to examine the effects of role (student vs. resident), BMI category (< 25 vs. >=25), and their interaction on total REAP-S scores. The total mean REAP-S scores for students was significantly higher (mean = 30.5, SD = 3.9) than that for residents (mean = 28.6, SD = 3.9; *p* = 0.006; Tables 1 and 2). Additionally, the total mean REAP-S scores were significantly higher for those with BMI < 25 (mean = 30.8, SD = 3.7) than for those with BMI > = 25 (mean = 28.3, SD = 4.0; *p* < 0.001). However, there was not a statistically significant interaction between role and BMI category on total REAP-S scores (*p* = 0.39). Thus, the total REAP-S scores between normal/low (< 25) and overweight/obese ( > = 25) BMI groups does not differ based on whether the participant was a student or a resident. The power for the test of the interaction was 0.14; the study may have been underpowered to detect a significant interaction.

**Table 1:** Mean REAP-S scores by role, BMI category

	Students		Residents		Total	
	Mean	SD	Mean	SD	Mean	SD
**BMI < 25**	31.6	3.6	29.5	3.5	30.8	3.7
**BMI > = 25**	28.9	3.9	27.8	4.1	28.3	4.0
**Total**	30.5	3.9	28.6	3.9	29.7	4.0

**Table 2:** P-values, effect sizes and power – REAP-S scores by role, BMI category

	p-value	Partial η2	Observed power
**BMI Category (low vs high)**	< 0.001	0.08	.97
**Role (student vs resident)**	0.006	0.04	.79
**Role x BMI Category Interaction**	0.390	0.00	.14

**Fig. 1:**
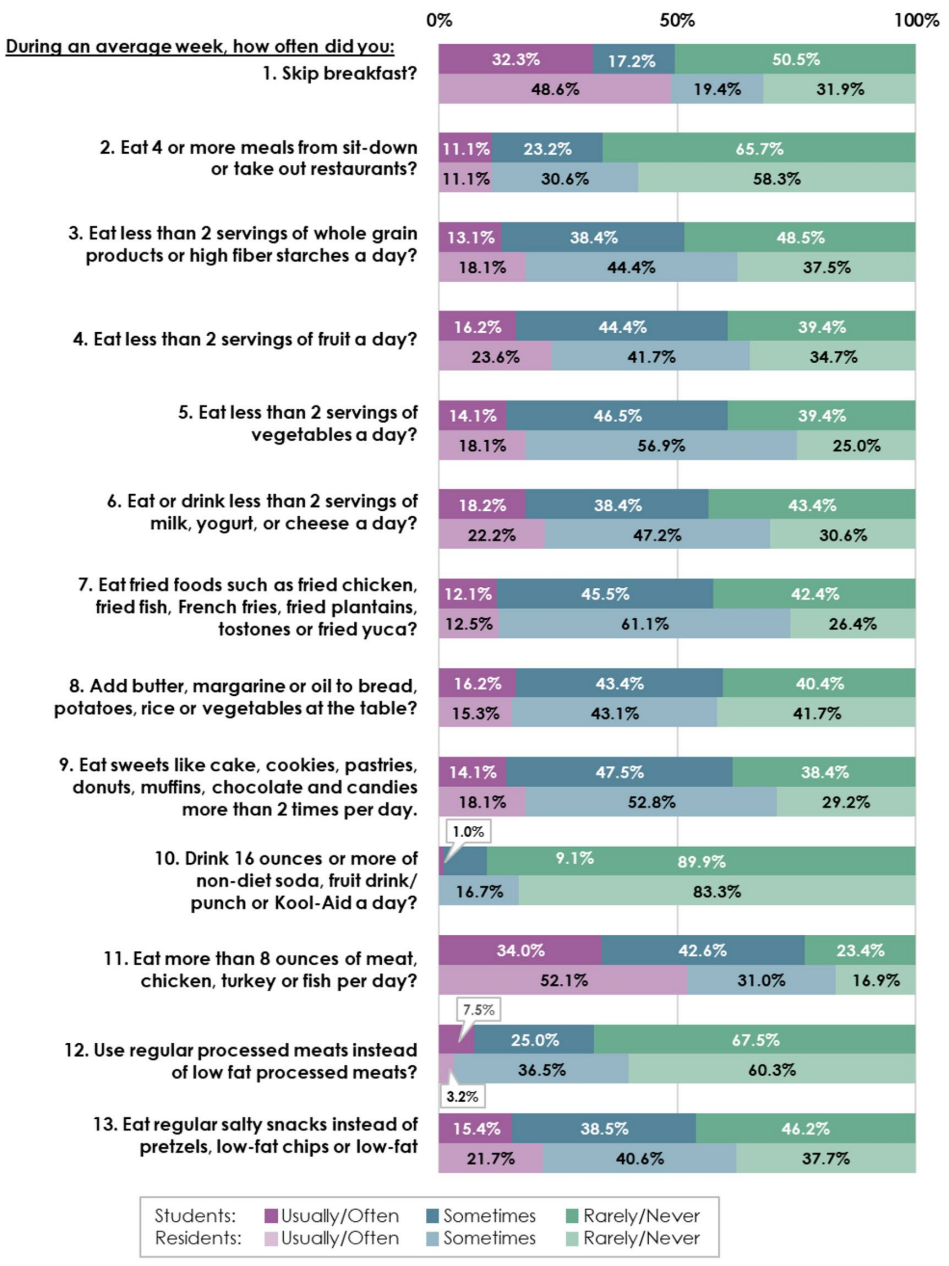
REAP-S question scores

Results of Chi-Square Tests of Independence or Fisher’s Exact Tests revealed that students and residents did not differ significantly (all p-values > 0.05) on all except one of the 13 individual items that comprise the total REAP-S scores. However, students trended toward healthier eating options (more frequently reporting “Rarely/Never” exhibiting poor diet quality behaviors; Fig. 1). A significant difference was found in the frequency with which participants skipped breakfast; 50.5% of students reported “Rarely/Never” skipping breakfast, significantly more than the 31.9% of residents who reported the same (*p* = 0.04).

Analyses of the additional unscored REAP-S questions assessing cooking and eating habits and willingness to change indicate that students reported a greater likelihood to prepare meals at home rather than eating restaurant meals (92.9%) when compared to residents (87.5%). However, this difference is not statistically significant (Table 3). Students reported a significantly higher likelihood of feeling well enough to shop or cook (96.0%) than residents (86.1%; p = 0.02). A combined 153 students and residents (89.3%) indicated that they would be “Somewhat” or “Very” willing to change their eating habits in order to be healthier.

**Table 3:** Unscored REAP-S item responses for students and residents

		Students		Residents		Total		
		#	%	#	%	#	%	p-value
**You or a member of your family usually shops and cooks rather than eating sit-down or take-out restaurant food?**	No	7	7.1%	9	12.5%	16	9.4%	0.23^a^
	Yes	92	92.9%	63	87.5%	155	90.6%	
**You or a member of your family usually feels well enough to shop or cook.**	No	4	4.0%	10	13.9%	14	8.2%	0.02^a^
	Yes	95	96.0%	62	86.1%	157	91.8%	
**How willing are you to make changes in your eating habits in order to be healthier?**	Very/Somewhat Unwilling	7	7.1%	1	1.4%	8	4.7%	0.01^b^
	Neither Willing Nor Unwilling	2	2.0%	8	11.1%	10	5.8%	
	Somewhat/Very Willing	90	90.9%	63	87.5%	153	89.5%	

^a^Analyzed using Chi-Square Test of Independence

^b^Analyzed using Fisher’s Exact Test

## Discussion

This study reports on the diet quality among a group of US medical students and primary care residents. On a scale of 13–39, where higher scores indicate better diet quality, 32 is the mean REAP-S score for adults eating a typical omnivorous diet; this has been recommended as a cutoff for comparison [2, 14]. Our overall sample had a mean REAP-S score of 29.7, confirming that the diet quality of these medical professional trainees is below average. Our findings are consistent with an overall trend in the US of a decline in diet quality [15, 23, 25–27].

We found that students had significantly higher total REAP-S scores, and thus better self-reported diet quality, than residents, supporting our first research hypothesis. Additionally, those with lower BMIs had higher REAP-S scores than those with higher BMIs, confirming our second hypothesis. There was not an interaction between role (student vs. resident) and BMI on diet quality, as predicted by our third research hypothesis.

Diet quality can be influenced by multiple systematic, individual, and local variables, including cultural and food environments, sociodemographic factors, and insomnia [27, 28]. There are several factors which may explain differences in overall REAP-S scores between students and residents, and to lower-than-average overall REAP-S scores for both groups.

Poor sleep may contribute to poor diet quality in healthcare students and residents. Sleep regularity has been found to be one of the most important variables related to food intake in a study of adolescents who are overweight or obese, and shorter sleep duration was directly correlated to lower total REAP scores and higher calories and fat intake [29]. Medical students have documented higher magnitudes of sleep deprivation compared to the general population [30]. Additionally, one study found that a shortened sleep cycle was associated with poor diet quality in medical students [31]. Residents likely face an even greater sleep deficit than medical students, with shift work and night calls occurring much more frequently [30, 32, 33]. This may explain, in part, their lower REAP-S scores.

Our data collection occurred during the COVID-19 pandemic (2020–2022), which has been shown to have a detrimental influence on diet quality [15]. A recent study found that several factors contributed to lower diet quality during the pandemic, including decreased time for food preparation and lower interest in healthy eating as well as increased frequency in eating away from home, higher food insecurity, increased anxiety, depression or boredom, and stockpiling of junk food [34]. Generally, the experiences of medical students and residents during the pandemic were vastly different, perhaps leading to differences in effects on diet quality. Students transitioned to distance learning, providing them with greater opportunity to prepare food at home. At the same time residents’ work schedules remained the same or became more intense.

Stress may be another contributing factor. The role of stress in diet quality is well-established, and medical students routinely experience higher stress levels than the same-age non-medical peers [35, 36]. Stress has been found to have been exacerbated during the pandemic in students [28, 34, 37, 38]. Moreover, residents have experienced more occupational stress during the pandemic than at any other time in the history of the medical profession [35, 36, 39].

The only specific REAP-S item on which our students and residents differed significantly was skipping breakfast, a factor that, in and of itself, has been found to contribute to poorer diet quality [38]. Residents reported “Rarely/Never” skipping breakfast at a much lower proportion than students. This finding corresponds to a previous study that found that residents increased the frequency with which they skipped breakfast in their first year of residency [40]. Differences in breakfast consumption between students and residents may contribute to the overall difference between residents and students on the total REAP-S scores.

A particular strength of our study was the use of the REAP-S, which has been validated in medical professional trainees and used extensively in other populations. Moreover, the REAP-S has recently been selected by the American Heart Association as one of three rapid diet assessment screening tools for cardiovascular disease risk reduction that are optimal for use in a clinical setting [9]. Previous studies with medical students that have reported on dietary and lifestyle practices [20] provided information on daily caloric intake and specific food intake but did not report on comprehensive dietary quality, [16, 21] whereas the REAP-S has been shown to correlate with cardiometabolic health of the surveyed population.

Our study has limitations inherent in any survey study examining self-reported data. The sample in this type of study is self-selecting, introducing possible bias. Data were collected from one midwestern institution, and the response rate was smaller than desired. Results may not be generalizable to other student and resident populations. Additionally, while students were surveyed at the height of the COVID-19 lockdown (June, 2020), residents were surveyed a year later, following the release of the COVID-19 vaccine. This may have contributed to group differences in weight and dietary behaviors. Another limitation is that we did not include measures of sleep, stress, and other possible pandemic-related factors such as change in work schedules, loss of loved ones, loneliness, food insecurity, that may be related to diet quality [41].

### Implications for research and practice

There are few precedent comprehensive studies in the US on medical trainee diet quality for comparison, so the role of sleep and stress, or other possible drivers of poor diet quality, cannot be teased out in this cohort. Future studies should examine the impact that poor sleep quality and increased stress have on diet quality in a cohort of medical trainees. Newer research in this area should use the updated REAP-S v.2 [42].

The overwhelmingly positive response to our question on the willingness to make changes in eating habits to be healthier is an encouraging finding. Reducing meals away from home and increasing nutrition knowledge and beliefs are associated with improved diet quality [43]. Recent evidence suggests that even brief nutritional training can increase medical students’ nutrition awareness [16]. Several such programs, such as Nutrition in Medicine, Healthy Kitchen, and Culinary Medicine, have been demonstrated to be helpful [44–47]. Future research should more closely examine how these efforts are related to improved diet quality in trainees. Because we collected our data during the COVID-19 pandemic, additional research is also needed to see if our results are replicated under more normal conditions.

Nutritional training has also been found to improve physician self-efficacy in dietary counseling for patients [48]. As trainees move into practice, an emphasis on nutrition education may translate into better nutrition counseling for their patients. Future studies could examine whether familiarizing medical trainees with additional screening tools may result in better nutritional care for patients. The REAP-S, accompanied by the WAVE, a tool that facilitates dialog between physicians and patients on Weight, Activity, Variety (of foods), and Excess (consumption), contain specific details on how diet quality can be improved and are designed to be used in clinical settings [49].

Less-than-optimal diet quality in our medical trainees may have significant long-term consequences. Poor REAP-S scores and increasing weight have been found to be correlated with cardiometabolic abnormalities, suggesting the potential for long-term adverse health outcomes in our study cohort [23]. However, the willingness to improve diet quality in our trainees affirms the potential for improvement. Longitudinal assessments and targeted nutritional education interventions may help maintain and improve the health of our future healthcare workforce.

Our study provides a quantitative assessment of the diet quality of medical students and primary care residents at an academic medical center in the midwestern United States. Both primary care residents and medical students had lower-than-average diet quality, with residents having lower quality when compared to medical students. We advocate for nutritional education, self-practice skills, wellness efforts, and sleep hygiene across the medical education continuum to improve diet quality for our future workforce. Ultimately, healthy providers will be influential in improving the diet quality of their patients and the health of the nation to mitigate the ever-increasing burdens of chronic care.

## Data Availability

Research data has been deposited to the Harvard Dataverse and can be accessed via 10.7910/DVN/AF1YVM.
